# Favourable changes in economic well-being and self-rated health among the elderly

**DOI:** 10.1016/j.socscimed.2011.02.027

**Published:** 2011-04

**Authors:** Gilbert Brenes-Camacho

**Affiliations:** Centro Centroamericano de Poblacion, Universidad de Costa Rica, San Pedro de Montes de Oca, San Jose 2060, Costa Rica

**Keywords:** Costa Rica, Transfers, Self-rated health, Socio-economic status, Quasi-experiment, The elderly

## Abstract

Adverse economic shocks exert an influence on health perceptions, but little is known about the effect of sudden positive changes in a person’s financial situation on self-rated health, particularly among low income people. This paper explores the association between an increase in the amount of non-contribution pensions, public cash transfers given to Costa Rican elderly of low socio-economic status (SES) and changes in self-rated health over time. The analysis is based on data from CRELES, the “Costa Rican Study on Longevity and Healthy Aging”, which is based on a probabilistic sample of people born in 1945 or earlier, and living in Costa Rica by 2002. The fieldwork for the first and second waves of CRELES was conducted from 2004 to 2006, and from 2006 to 2008, respectively. The Costa Rican Government raised the amount of the non-contribution pension for the poor 100% before July 2007, and an additional 100% after that date. Due to the CRELES fieldwork schedule, the data have a natural quasi-experimental design, given that approximately half of CRELES respondents were interviewed before July 2007, independently of their status in receiving the public cash transfers. Using random effects ordered probit regression models, we find that people who experienced such increase report a greater improvement in self-rated health between waves than those who experienced a smaller increase and than the rest of the interviewees. Results suggest that increases in income may lead to a greater improvement in self-rated health.

## Introduction

The socio-economic status (SES) gradient in health and mortality refers to health differentials by income, education, occupation, and social class: People with higher SES have better health and lower death rates (Kawachi, Adler, & Dow, 2010; Macintyre, 1997; Marmot, 1994; Preston & Taubman, 1994; Smith, 1998; Smith & Kington, 1997). SES can impact health through availability of resources to purchase and manage medication and health services, as well as healthier goods and lifestyles, and better education may help in adopting healthy practices and enhances the ability to understand and deal with the complexities of health services. SES can also influence health through relative levels of income or wealth that a person has rather than through the absolute amount of economic resources, or through the relative social position (ranking) of a person with respect to others (Kawachi, Adler, & Dow, 2007; Marmot et al., 1991).

Among some elderly populations (e.g., in the U.S.), the SES gradient in health appears less steep than among younger groups (Deaton & Paxson, 1998; Smith, 2004); it might hold only for certain conditions (notable, mental disorders and self-rated health), but not for others; or it might have the opposite direction to what is expected (Adams, Hurd, McFadden, Merrill, & Ribeiro, 2003; Rosero-Bixby & Dow, 2009). However, changes in pension income, especially among poorer populations, may have a strong impact on health (Case, 2001; Duflo, 2003; Jensen & Richter, 2004).

Empirically, it is difficult to determine the direction of the causal relationship between health and SES components, such as education, income, and wealth. The association might be due to reverse causation or to an omitted variable bias (Kawachi, Adler, & Dow, 2007; Smith, 2004). Natural experiments and quasi-experiments have also been used to control for reverse causation and omitted variables. These analyses rely on unexpected, sudden, and possibly large income changes, and compare the health status of those who experience the changes versus those who do not experience them. Some of these studies find that income increases or losses are related to some health indicators, but not to all (Apouey & Clark, 2009); mental health and self-rated health are linked to income shocks in several of these studies (Frijters, Haisken-DeNew, & Shields, 2005; Gardner & Oswald, 2007). Several studies have analyzed the relationship of public cash transfers to the poor with household health using natural quasi-experimental designs given that they compare persons or households who experienced changes in pension income against those who did not. These quasi-experimental studies have analyzed how cash transfers or changes in pensions affect health. In the U.S., Snyder and Evans (2006) found that higher pensions lead to higher mortality because persons eligible for higher income did not engage in more employment. The quasi-experimental studies in Mexico (Gertler, 2004) and South Africa (Case, 2001; Duflo, 2003) have found that cash transfers are beneficial to child health. In Russia, Jensen and Richter (2004) found that pension loss increased mortality.

Self-rated health has been used as a summary measure in studies about the SES gradient of health, including some studies mentioned above (Adams et al., 2003; Cheng et al., 2002; Frijters et al., 2005; Monden, 2005; Smith, 2004; von dem Knesebeck et al., 2003). Self-rated health is a good predictor of mortality (Idler & Benyamini, 1997) and health services utilization (Dening et al., 1998). It is associated with disease burden and physiological markers of health (Goldman, Glei, & Chang, 2004; Lee & Shinkai, 2003). It is also influenced by psychological characteristics such as sense of control, sense of well-being, life satisfaction, behavioral intentions on health improvement, positive and negative affect, and depression (Bailis, Segall, & Chipperfield, 2003; Benyamini, Idler, Leventhal, & Leventhal, 2000; Bobak, Pikhart, Hertzman, Rose, & Marmot, 1998; Lee & Shinkai, 2003; Schneider et al., 2004), and by the socio-economic context in which the person lives (Kawachi, Kennedy, & Glass, 1999). Hence, life stressors can influence the way people rate their own health.

This article intends to explore how increases in income among the destitute can improve the way people rate their own health. After a means test, the Costa Rican government provides free access to public health care services and monthly cash transfers, called non-contribution pensions, to low SES elderly. The Arias Sanchez administration (2006–2010) decided to raise the amount of money paid through the non-contribution pension system. After the presidential inauguration, the cash transfers were raised from 16,000 colones (approximately US$32) per month to 35,000 colones (approximately US$70) per month: close to a 100% increase. The non-contribution pensions were raised again to 50,000 colones (approximately US$100) after July 2007: almost 200% increase from the amount paid during 2005. The analysis takes advantage of this natural experiment design. Therefore, approximately half of the survey respondents entitled to these public cash transfers experienced the 100% increase between the first and the second waves, while the other half experienced the 200% increase. The survey also asks respondents to rate their own health in both waves. Therefore, the article studies whether the people entitled to such public transfers and interviewed after July 2007 rate their health better on average, than people interviewed in July 2007 or before, controlling for confounders.

Aside from non-contribution pensions, around half of the Costa Rican elderly population receives a retirement pension, called a contribution pension because formal workers have to make mandatory contributions to the main public insurance and pension fund through payroll deductions and mandatory contributions from employers and the state. The main public fund is administered by Caja Costarricense del Seguro Social (CCSS, the Costa Rican Social Security Fund). This main public fund is a pay-as-you-go system. Widows and young children are automatically entitled to the contribution pension after the beneficiary’s death; this is the so-called “inherited pension”. Some elderly people may not be retired yet and still be contributing to the Social Security fund through payroll deductions if they have not made enough contributions. These workers and their family are entitled to the public health insurance.

## Objectives

The general goal of this paper is to study how income increases influence self-rated health. More specifically, the main objective is to determine whether a substantial rise in non-contribution pensions to poor elderly in Costa Rica made them improve the rating of their own health.

## Methods

We use the dataset from CRELES, the “Costa Rican Study on Longevity and Healthy Aging”. It is an ongoing longitudinal study of a nationally representative sample of 2827 adults born in 1945 or before (ages 60 and over at the first interview) and residing in Costa Rica by the year 2000. CRELES has been approved by the Institutional Review Board (Comité de Ética) of the University of Costa Rica. The first wave of interviews was conducted from November 2004 through August 2006. The second wave started in November of 2006 and concluded in July 2008. The description of the fieldwork, and the collection and processing of specimens can be found in Mendez-Chacon et al. (2007).

CRELES has a complex sampling design. There is an original master sample of 9600 individuals that was randomly selected from the 2000 census database with stratification by 5-year age groups and over sampling of older individuals. Within each stratum, persons were selected using simple random sampling involving a systematic selection procedure. In the master sample, sampling fractions ranged from 1.1% among those born in 1941–45 to 100% for those born before 1905. The individuals in the master sample were grouped into 102 geographical clusters according to the 102 “Health Areas” created by the Government. The final sample for the in-depth interview is composed of a probabilistic sub-sample of clusters: 60 “Health Areas” (out of a total of 102). This sub-sample originally included nearly 5000 individuals and covered 59% of Costa Rican territory. The first wave fieldwork yielded the following non-response rates: 19% deceased by the contact date; among those alive, 18% were not found in the field, 2% moved to other addresses, 2% rejected the interview, and 2% were not found after several visits (likely rejections). After non-response, the resulting sample size for the first wave amounts to 2827 individuals. Similar non-response rates were found for the second wave. All statistical analyses take sampling weights (inverse of selection probabilities) into account. Furthermore, 703 persons in the first wave and 676 in the second wave needed a proxy respondent to answer the survey questions. Given the focus of this analysis on perceptions, we exclude proxy respondents. We also exclude respondents who died before the first wave, who needed a proxy respondent in at least one wave, or who had missing values in any of the covariates of the regression models. The models are estimated using only 1556 respondents, which correspond to 55% of the first wave total sample size and 66% of the second wave total sample size. Given that we assume that self-rated health is a subjective measure determined by the awareness of the respondent to his or her socio-economic condition, the selected sub-sample of the analysis should not be seen as a problem but as the desired sub-sample to draw conclusions on.

## Variables

Self-rated health is the main dependent variable. The variable comes from the answers to the question “How would you say your health is now: Excellent, Very Good, Good, Fair, Bad?” The same question was asked in both waves. The variable was coded as 5 for excellent, 4 for very good, 3 for good, 2 for fair, and 1 for bad, so that positive values in regression coefficients mean an improvement in perceptions of own health.

A series of other health and perception variables are used as complementary dependent variables. We choose variables that can change in a short period of time: disability (dichotomous, having at least one limitation in Activities of Daily Living ADL or Instrumental Activities of Daily Living IADL), depression (dichotomous, having 9 or more out of 15 Yesavage symptoms of geriatric depression, Rosero-Bixby & Dow, 2009), satisfaction with life (scale of 4 categories from unsatisfied to satisfied), systolic and diastolic blood pressure, C-reactive protein, High Density Lipoprotein HDL, total serum cholesterol, glycated hemoglobin HbA_1C_, and triglycerides.

We would expect that, if the relationship between self-rated health and income is mediated by mental health, we would find significant effects of the 200% increment in non-contribution pensions for depression, satisfaction with life, and potentially systolic and diastolic blood pressure. CRELES does not have longitudinal information for stress biomarkers. Significant effects for the other allostatic load biomarkers, such as CRP, HDL, cholesterol, triglycerides and HbA_1C_ (Seeman, McEwen, Rowe, & Singer, 2001), might indicate that the processes linking self-rated health and changes in income are related to other health factors.

As mentioned before, the analysis can be treated as a natural quasi-experiment because only one sub-group as a whole experienced a 200% increase in their fixed income: respondents who are entitled to a non-contribution pension and were interviewed after July 2007 during the second wave. This population can be considered as the main “experimental group”. There is a secondary “experimental group” that is composed of contribution retirement pension earners who were interviewed after July 2007. This is a secondary “experimental group” because they did get an increase in their pension money between wave 1 and wave 2: an approximate rise of 42% (Programa Estado de la Nacion en Desarrollo Humano Sostenible, 2009). Costa Rican law establishes that the minimum retirement pension has to be raised as non-contribution pensions are raised. This increase is the reason why contribution pension earners (retirees) cannot be considered as the “control group”.

The “control group” can be defined in two different ways. If only non-contribution pension earners are analyzed, the control group is determined by those pensioners interviewed in the second wave before July 2007, while the experimental group is composed of pensioners interviewed after June 2007. The sub-sample size for this control group is 149, while for the experimental group is 136. Notice that in this analysis, the sample size is relatively small, therefore the statistical power for the regression coefficient is smaller. In this sense, among contribution pensioners (retirees), the sizes of the control and experimental groups are 355 and 315.

If the whole elderly population (who did not need proxy respondents) is analyzed, then the definition of the control group is more complex because it is composed by three different groups:

(a) respondents entitled to a non-contribution pension and were interviewed before the change in July 2007 during the second wave,
(b) respondents entitled to a retirement pension and were interviewed before the change in July 2007 during the second wave, and
(c) the rest of the elderly population.

The size of this control group is 1102 individuals. There are two advantages in analyzing the whole elderly population instead of only the control group. The main advantage is that comparing the experimental groups with the rest of the elderly population helps in controlling other period effects. If health is better rated not only by non-contribution pension earners interviewed after June 2007, but also by the rest of the population, then this result would suggest that there is another factor with an incidence on self-rated health aside from the changes in the non-contribution pensions. The other advantage is enhanced statistical power.

Given the definitions of the experimental and control groups, three main explanatory variables are constructed: (a) a dummy variable on whether the individual was interviewed before or after July 2007, (b) an interaction variable between this dummy variable and being a contribution pension-earner, and (c) another interaction variable between the first dummy variable and being a non-contribution pension earner. The role of these dichotomous variables is explained in the description of the regression model, below.

These dichotomous variables are the main explanatory variables. The analysis controls for several confounding variables: age, being female, living in Metropolitan Area and in urban areas, marital status (divided in three dummy variables: being married or cohabiting, being widowed, and others, which is the reference category), education (having less than 6 years of schooling), and self-reported economic situation, which is a scale similar to the self-rated health scale. Additionally, there is a dichotomous variable for every self-reported disease diagnosed by a physician: hypertension, hypercholesterolemia, diabetes mellitus, cancer (except skin cancer), chronic pulmonary disease, heart attack, other heart diseases, stroke, arthritis, and osteoporosis.

## Methods

Two random-effects ordered probit regressions are used to estimate the effect of the dummy variables that define the experimental and control groups on self-rated health. In the analysis of the whole elderly population, the model can be represented by the following equations:

(1) $$z_{itj}=\gamma d_{1it}+\delta d_{2it}+\lambda d_{3it}+\beta_{0}+\beta_{1}x_{1it}+\ldots +\beta_{k}x_{kit}+a_{i}+u_{it}$$

(2) $$P\left(y_{tj}=j\right)=P\left(\kappa_{j-1}<z_{itj}<\kappa_{j}\right)=\Phi \left(\kappa_{j}-z_{itj}\right)-\Phi \left(\kappa_{j-1}-z_{itj}\right)$$

The *t* sub-index refers to the survey wave (1,2), the *i* sub-index refers to the respondent, and the *k* sub-index refers to the number of confounding variables. $\Phi \left(z_{itj}\right)$ is the standard normal cumulative probability function, and $\kappa_{j}$ and $\kappa_{j-1}$ are cutoff points needed to classify the linear combinations into the *j* category of the ordered variable. The term *a_i_* corresponds to the random effect of each individual *i*, while *u_it_* is the random disturbance term. The variables *d*_1*it*_, *d*_2*it*_ and *d*_3*it*_ are the dummy variables for being interviewed before or after July 2007 (*d*_1*it*_), the interaction with non-contribution pension-earners (*d*_2*it*_), and the interaction with retirement pension-earners (*d*_3*it*_). We say that there is an effect of the rise of non-contribution pensions on self-rated health if δ (the coefficient for *d*_2*it*_) is positive and significant, while γ (the coefficient for *d*_1*it*_) is not significantly different to zero. If *γ* is significant and has the same direction as any of the interaction variables, we can argue that changes in self-rated health are due to a contextual effect that affected the whole elderly population during the inter-wave period, rather than just the rise in pensions that benefited the people receiving public cash transfers. If *λ* (the coefficient for the interaction of time with retirement pension-earners) is significant and has the same direction as *δ*, it means that retirement pension-earners change their health ratings in the same way as non-contribution pension earners; thus, it would not be clear whether the change in self-rated health is due to the increase in the non-contribution pensions or to some other unmeasured variables.

In the analysis of only non-contribution pensioners, the model simplifies to:

(3) $$z_{itj}=\gamma d_{1it}+\beta_{0}+\beta_{1}x_{1it}+\ldots +\beta_{k}x_{kit}+a_{i}+u_{it}$$

The set [*x*_1it_, …, x_*kit*_] refers to confounders. Several equations are estimated sequentially. Model 1 only controls for pension status (the main binary covariates); model 2 adds socio-demographic variables (sex, age, marital status, and education) and morbidity; model 3 adds self-rated economic situation. We use several significance level: 0.05 and 0.01 for the whole elderly population; 0.10 in the analysis for non-contribution pension earners only, given the limited statistical power.

In order to explore the mechanisms that might link self-rated health with changes in income, we also estimate models for each complementary health variable: ordered probit for satisfaction with life; binary probit for being depressed and for having at least one ADL/IADL limitation; and a Gaussian linear model for systolic and diastolic blood pressure, HDL, total cholesterol, triglycerides, HbA_1C_, and CRP; the last two are logged to control for the skewness of the variables distribution. All of these models are random-effects models to control for longitudinal data. The sample sizes for these models are smaller than the sample sizes for the previous models given that several biomarkers have more missing values.

## Results

Before estimating the model that tests the effect of the rise in non-contribution pensions on self-rated health, it is necessary to describe the sample that is being analyzed. Table 1 summarizes the characteristics of the elderly who did not need proxy respondents and gave answers in both waves. In general, people became more ill throughout the two-year inter-wave period. The largest increase in diagnosed disease prevalence is observed in hypertension, hypercholesterolemia, other heart diseases (different to heart attacks), and diabetes mellitus. Table 2 describes the inter-wave differences in the additional health variables (including biomarkers). Diastolic blood pressure and total cholesterol were lower in wave 2, while glycated hemoglobin was significantly higher.

Regarding Social Security status (Table 3), only 5% of the sub-sample reported being uninsured; 39% had some sort of insurance but no pension, and the final 56% had health insurance and earned some kind of retirement pension: 44% in the second wave were entitled to a retirement pension because they contributed to the system while working or because they inherited it from a family member (typically, the spouse) who died. Additionally, 12% reported having a non-contribution pension in the second wave. Roughly half of non-contribution pension earners were interviewed for the second time before July 2007 and the other half from July 2007 on. Notice that the median non-contribution pension amounted to 16,000 colones during the first wave, to 35,000 before July 2007, and to 50,000 colones after that month.

Notice how the control and experimental groups are formed based on the distribution in Table 3. Table 4 describes the relative distribution of the categories of self-rated health for the two definitions of control and experimental groups, as well as for the total population. Forty six percent of respondents report to have fair or bad health in wave 1; this proportion decreased to 41% in wave 2. The inter-wave change in the distribution is not significant at a 0.05 level, but it is at a 0.10 level. This proportion is very similar to those found in five cities of the Latin American SABE project (Wong, Pelaez, & Palloni, 2005). Notice that the distribution of self-rated health is concentrated in worse categories among non-contribution pension earners that were interviewed before July 2007, when compared to the extended control group that were interviewed during the same period. On the contrary, the distribution of non-contribution pension earners who were interviewed after June 2007 is more similar to the distribution for the extended control group interviewed during the same period. This suggests that the change in pensions might have made self-rated health of poor elderly resemble the distribution of the rest of the population.

The random-effects ordered probit regressions are used to control for confounding effect in the relationship between Social Security status and self-rated health (Table 5). The first set of models that is presented is estimated with non-contribution pensioners only. The effect is measured with the coefficient for the dummy variable that differentiates the time of interview (γ). The coefficient is significantly different to zero at an *α* = 0.10. Its size remains basically unchanged after adding control covariates. The sign of the coefficient is positive, which means that non-contribution pension earners interviewed after June 2007 rated their health better (on average) during the second wave than those interviewed before July 2007. The conclusions are similar if the complete elderly population is analyzed (bottom part of Table 5). The negative and significant coefficient for the dichotomous variable “non-contribution pension-earner” indicates that, on average, this group of elderly Costa Ricans report worse self-rated health than the other groups. The non-significant coefficient for the time variable “Interviewed after July-07” (the γ term described in the Methods section) suggests that there is no change in self-rated health for the total population among those interviewed after June-07; however, the coefficient for the interaction between these two variables –non-contribution pension-earners and the time variable– (the δ term) is significant at a 0.05 level. The size of the coefficient remains after controlling for the control covariates. These estimates agree with the results in the previous set of models that were restricted to non-contribution pensioners only. This expanded equation provides a more thorough picture because it shows that the improvement in self-rated health among non-contribution pensioners interviewed after June 2007 is not shared by the rest of the elderly population.

Additional models are estimated in order to analyze the effect of the pension changes on other health variables (Tables 6 and 7). According to the equations restricted to non-contribution pensioners, there is a decrease in diastolic blood pressure (but not in systolic blood pressure) among those interviewed after June 2007. However, when the total elderly population is analyzed, the models show that this change was generalized to all respondents interviewed after July-07 (γ = −1.678, *p* < 0.01), and not just to non-contribution pensioners interviewed during that time (δ = −0.095, *p* > 0.10). There are similar findings with hypercholesterolemia biomarkers (Table 7). In the restricted sub-sample, non-contribution pensioners interviewed after June 2007 had on average higher HDL levels and lower total cholesterol levels; however, the analysis with the total sample show that this changes are observed for the total elderly population interviewed after June 2007, and not only for non-contribution pensioners. The models do find a result that is contradictory with an improvement in self-rated health: people who got the 200% increase in non-contribution pensions report on average a higher probability of having ADL/IADL limitations (δ = 0.374, *p* < 0.10) than the rest of the respondents (Table 6). Including any of these health variables as confounders in the main equation for self-rated health does not change the significant coefficients for the experimental group.

**Fig. 1:**
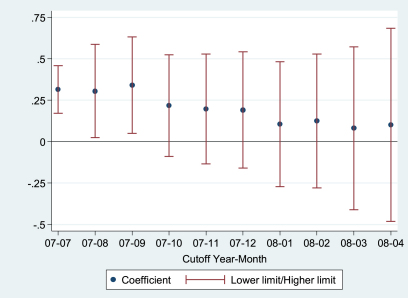
Ordered probit regression coefficient for the interaction between time of interview and non-contribution pension earner, with varying month in the cutoff point for the time of interview (95% Confidence Interval).

As explained before, the most important effect in the analyses is tested with the coefficient *δ* for the interaction between the time dummy variable and the dummy variable that refers to receiving a non-contribution pension in the equation about self-rated health. The time dummy variable is equal to 1 if people were interviewed after June 2007, and is equal to zero otherwise. In order to test the robustness of this operationalization and explore how immediate the effect happened after the change was applied, we re-estimate the random effects ordered probit model several times changing the operationalization of the time dummy variable. Instead of July 2007, we use more recent months as cutoff points: August 2007, September 2007, etc. If the coefficient *δ* increases when the cutoff point is postponed, this means that there is a lag in the effect of the rise of non-contribution pensions. If the size of *δ* remains roughly the same after the cutoff point is postponed some months, this means that the effect of the change in the public cash transfers on self-rated health occurs almost immediately after the change started to apply. According to Fig. 1, the value of the coefficient *δ* remains roughly the same despite the change in the cutoff month. The coefficient is no longer significant (given that the confidence interval includes the zero value) when the cutoff month is October 2007 or more recent, but this is mainly due to lack of statistical power because the size of the coefficient remains roughly the same, at least until December 2007 is used as the cutoff point.

## Discussion

Based on this analysis, an increase in income is positively associated with an increase in average self-rated health, and the amelioration in perceived health seems to occur almost immediately after the raise in income takes place. The analyses do not clearly explain the mechanism behind this association. The immediacy of the effect suggests that psychological mechanisms might be the ones explaining the observed relationship: people who got a larger relative increase in their income (through the non-contribution pension) might feel less stressed due to their socio-economic situation and therefore rate their health better than those who received a smaller increase or no increase at all.

The psychological explanation seems more likely than the resource-availability explanation given the Costa Rican context. All of the Costa Rican elderly receiving the non-contribution pension are also entitled to free health insurance, and the public health care system is still the most widely used in Costa Rica, especially among the elderly (Brenes-Camacho & Rosero-Bixby, 2008). This plausible explanation is in agreement with several authors who analyze the psychological dimensions of self-rated health (Benyamini et al., 2000; Bobak et al., 1998; Lee & Shinkai, 2003; Schneider et al., 2004). However, CRELES data do not have information about stress level to test whether this explanation is plausible.

The analyses tries to control for psychological dimensions by including a binary variable about depression based on the Geriatric Depression Scale, and an another self-reported scale about satisfaction with life. Controlling for these variables does not change the main results. The new stage of the CRELES study is collecting stress information with a battery of questions, but the original CRELES dataset (the one used for this article) does not allow a clear description of this causal mechanism. The analyses neither show that other biological mechanisms, especially biomarkers related to the allostatic load concept, are mediating the relationship.

If the stress explanation is true, these results have several implications for the observed association between income and self-rated health. Relieving the poor from socio-economic stressors –even partially– may ameliorate a general sense of healthiness among the population and may reduce the steepness of the SES health gradient. Generous welfare systems might then have an impact in improving population health not only by providing health care resources to the poor but also by leveling off stress derived from an unstable economic situation.

These optimistic results might be affected by several limitations in the analyses. The most important limitation is that the increase in non-contribution pensions does not yield an exact “natural experiment” design. The “experimental” and “control” groups were not observed simultaneously with a “natural” random allocation of “treatment” and “control” to the experimental units. This “natural quasi-experiment” design resulted from a particular trait of CRELES fieldwork strategy: its fieldwork takes place along a two-year period. The observed significant coefficient in the model might be due to any other unobserved factor that occurred after July 2007, especially among non-contribution pension-earners. We aimed to control this effect by including the rest of the whole population as an additional “control” group and by including possible confounders in the models. However, there might still be some confounding effect that could not be controlled for.

The most important source of non-randomness in the allocation of respondents into the experimental and control groups is time. Respondents in the control group were interviewed during the first part of the second wave, while the experimental group was interviewed during the second part. Important changes in health can happen during a year of their life, especially, among the eldest. As an example, the analysis of dependent health variables show that the prevalence of ADL/IADL limitations significantly increases among non-contribution pensioners interviewed after June 2007 (the experimental group). This is an expected result given that disabled elderly are a priority target for non-contribution pensions. Nevertheless, according to the study, these respondents rated their health better, even though they became more disabled. It is difficult to establish what other circumstances of the Costa Rican context might explain possible residual confounding. The “Costa Rican Report on the Situation of the Aged Person” (Fernandez & Robles, 2008) does not report any other remarkable change in the elderly population’s social, health, or economic context, except the increase in the non-contribution pension.

Finally, the Costa Rican Government decided to raise the amount of money provided through the non-contribution pension system during a period of fiscal expansion, but this fiscal expansion has been followed by the world-wide financial crisis of 2008/2009. Given that Costa Rica was affected by this financial crisis (CEPAL, 2008; Cordero, 2009), it is not clear whether the observed improvement in average perceived health among Costa Rican elderly can endure for long.

As a conclusion, these results have important implications for the Costa Rican context. The rise in the amount of money was aimed at decreasing the proportion of people living under the poverty line in Costa Rica (Fernandez & Robles, 2008). The results of these analyses suggest that the effectiveness of this policy transcended its main goal, having also an impact in the health perceptions of its target population. This conclusion also suggests that the good performance of Costa Rica in health indicators might be related to public policies aimed to the neediest segments of its population (Barahona Montero, 1999; Mesa-Lago, 1999, 2004, 2008).

These findings may have implications for other Latin American countries. For decades, few countries in the region have had non-contribution pensions (Mesa-Lago, 2008). These same countries have an advanced population aging process, as well as some of the best health indicators in the region. Empirical evidence shows that non-contribution pensions have helped in reducing the prevalence of poverty among the elderly in these countries. In recent years, other Latin American countries –chiefly, Bolivia and Ecuador– have successfully implemented this kind of public cash transfers (Mesa-Lago, 2008; Rofman & Lucchetti, 2006). Although Bolivia and Ecuador do not have as good health conditions as the first group of countries, the implementation of non-contribution pensions may help in improving them. Therefore, these results may imply that countries that keep improving or reviewing their non-contribution pension system may advance the welfare of their low SES elderly population, in addition to the reduction of poverty prevalence.

## Figures and Tables

**Table 1:** Costa Rica. Characteristics of respondents born before 1945, with a response in each wave, and with no need of proxy respondent, in wave 1 (2004–2006) and wave 2 (2006–2008) (Unweighted *n* = 1553 in each wave; 3006 observations).

Characteristics	Wave 1	Wave 2
Quantitative variables (mean ± s.d.)		
Age	68.4 (6.6)	70.2 (6.6)
Categorical variables (%)		
% Female	52.1	52.1
% in Metropolitan area	52.2	52.2
% Urban	63.1	63.1
% Married or cohabiting	64.8	62.6
% Widowed	17.9	19.7
% Others not in union	17.3	17.7

% less than 6 y of education	55.5	55.5
% fair/bad self-reported econ. situation	56.8	49.0

% with self-report of diagnosed:		
-Hypertension	46.7	54.2
-Hypercholesterolemia	41.3	53.0
-Diabetes mellitus	19.5	23.6
-Cancer	4.6	5.3
-Chronic pulmonary disease	15.3	16.9
-Heart attack	3.9	4.7
-Other heart diseases	10.8	15.0
-Stroke	1.9	2.2
-Arthritis	14.0	16.6
-Osteoporosis	9.3	12.0

**Table 2:** Costa Rica. ADL/IADL limitations, depression status, self-reported satisfaction with life, and mean levels (±s.d.) of biomarkers for respondents born before 1945, with a response in each wave, and with no need of proxy respondent, in wave 1 (2004–2006) and wave 2 (2006–2008) (Unweighted *n* = 1414 in each wave; 2828 observations).

Biomarkers	Wave 1	Wave 2	Tests for differences in paired samples^a^
%with at least one limitation in ADL or IADL.^b^ (Disability)	59.4	61.2	0.207
%with depression	8.8	9.2	0.764
Satisfaction with life (%)			
%Very satisfied	75.4	78.4	0.196
%Somewhat satisfied	20.4	17.7	
%Somewhat unsatisfied	3.0	3.2	
%Very unsatisfied	1.2	0.7	

Systolic blood pressure (mm/Hg)	143.39 (21.72)	143.20 (21.63)	0.681
Diastolic blood pressure (mm/Hg)	83.93 (11.62)	81.66 (11.84)	0.000
C-reactive protein CRP (mg/L)	0.57 (0.85)	0.48 (0.62)	0.731

High-density lipoprotein HDL (mg/dL)	43.96 (13.11)	42.98 (12.32)	0.012
Total cholesterol (mg/dL)	218.00 (49.07)	204.81 (41.66)	0.000
Glycated hemoglobin HbA_1C_ (%)	5.75 (1.17)	6.11 (1.21)	0.000
Triglycerides (mg/dL)	168.82 (92.50)	171.91 (84.97)	0.282

a McNemar test for depressed and disability; *X*^2^ symmetry test for satisfaction with life; paired *t*-test for biomarkers.

b ADL = activities of daily living; IADL = instrumental activities of daily living.

**Table 3:** Costa Rica. Social security status of persons born before 1945 in wave 1 (2004–2006) and wave 2 (2006–2008) (Weighted estimates).

Social security status	(*n*)	% Wave2 (weighted)	Median pension^a^	
			Wave1	Wave2
CONTROL GROUP				
No pension				
-Uninsured	60	4.7		
-Insured by contribution (or family)	489	36.0		
-Insured by the State	49	3.2		
Pension earners interviewed before July 2007 in wave 2				
-Non-contribution pension	149	6.4	16.0	35.0
-Retired by contribution	355	22.8	66.0	98.0
Total Control Group	1102	73.1		
EXPERIMENTAL GROUPS				
Pension earners interviewed in July 2007 or later in wave 2				
-MAIN: Non-contribution pension	136	5.9	17.0	50.0
-SECONDARY: Retired by contribution	315	21.0	90.0	109.0

a In thousand colones (current colones); 500 colones ≫ is approximately equal to US$ 1.00.

**Table 4:** Costa Rica. Relative distribution of self-rated health by experimental or control groups, in wave 1 (2004–2006), and in wave 2 (2006–2008) (Weighted estimates).

Social security status	Self-rated health					*p*-value of *X*^2^ test of symmetry for paired ordinal var *p*-value
	Bad	Fair	Good	Very good	Excel	
(unweighted *n* = 1553)						
TOTAL SAMPLE						
-Wave 1	6.0	40.5	33.4	12.6	7.5	0.0874
-Wave 2	4.7	36.7	38.3	13.3	7.0	

Extended control group						
-Int. before Jul-07 in w2:						
Wave 1	5.8	39.4	33.7	13.9	7.2	0.000
Wave 2	4.6	32.3	39.9	15.2	8.0	
-Int. after Jun-07 in w2:						
Wave 1	3.4	41.6	31.8	15.0	8.2	0.185
Wave 2	4.0	39.4	38.8	12.6	5.2	

Only pensioners control group						
*Non-contribution pension earners*						
-Int. before Jul-07 in w2:						
Wave 1	15.1	50.8	22.9	8.4	2.8	0.169
Wave 2	8.2	42.6	36.2	8.8	4.2	

*Contribution pension earners*						
-Int. before Jul-07 in w2:						
Wave 1	3.2	31.5	38.4	17.9	9.0	0.907
Wave 2	3.7	28.5	41.6	18.4	7.8	

Pensioners experimental groups						
*Non-contribution pension earners*						
-Int. after Jun-07 in w2:						
Wave 1	10.9	43.0	32.8	5.8	7.5	0.312
Wave 2	6.6	40.6	37.6	13.1	2.1	

*Contribution pension earners*						
-Int. after Jun-07 in w2:						
Wave 1	2.4	32.3	34.3	17.4	13.6	0.801
Wave 2	3.0	30.3	34.4	19.4	12.9	

**Table 5:** Coefficients of random-effects ordered probit regression for self-rated health^a^ on Social Security status variables and other covariates, in equations with sequential addition of control variables^b^^c^

Variables	Model 1	Model 2	Model 3
*Ordered probit regression with only non-contribution pension earners (Number of observations*=*570; number of individuals*=*285)*			
(Ref: Interviewed before July 2007)			
Interviewed after June 2007 (γ)	0.217 (0.121)†	0.278 (0.120)∗	0.222 (0.120)†
*Ordered probit regression with all elderly population (Number of observations*=*3106; number of individuals*=*1553)*			
(Ref: Other social security status)			
-Contribution pension-earner (CP)	0.218 (0.076)	0.096 (0.076)	0.051 (0.074)
-Non-contrib. pension-earner (NCP)	−0.513 (0.096)∗∗	−0.293 (0.093)∗∗	−0.258 (0.090)∗∗
Interviewed after June-07 (γ)	−0.062 (0.076)	−0.029 (0.076)	−0.058 (0.075)
(Ref: Interviewed before)			

INTERACTIONS:			
After June-07 X NCP			
(Non-contribution pension) (δ)	0.315 (0.148)∗	0.344 (0.145)∗	0.317 (0.144)∗
After June-07 X CP (Contribution pension) (λ)	0.139 (0.118)	0.112 (0.116)	0.123 (0.115)

a Self-rated health is coded as 5.Excellent, 4.Very good, 3.Good, 2.Fair, 1.Poor.

b †:*p* < 0.10; ∗:*p* < 0.05; ∗∗:*p* < 0.001.

c Model 1: Only variables defining experimental and control groups; Model 2: Model 1 + Sociodemographic variables + Health variables; Model 3: Model 2 + Self-rated economic situation.

**Table 6:** Coefficients of random-effects regressions for self-rated satisfaction with life^a^, being depressed according to the Yesavage scale of Geriatric Depression^b^, having at least one ADL/IADL limitation (Disability)^b^, systolic and diastolic blood pressure^c^, on social security status variables and control covariates.

Variables	Ordered probit	Binary Probit	Binary Probit	Linear (Gaussian)	Linear (Gaussian)	
	Satisf. with life	Depressed	ADL/IADL limitations	Systolic BP	Diastolic BP	
*Ordered probit regression with only non-contribution pension earners (Number of observations*=*464; number of individuals=232)*						
Interviewed after June 2007	−0.041 (0.181)	0.163 (0.325)	0.229 (0.190)	−2.613 (2.205)	−2.900 (1.202)∗	
*Ordered probit regression with all elderly population (Number of observations=2708; number of individuals=1359)*						
(Ref: Other social security status)						
-Contribution pension-earner (CP)	0.118 (0.123)	0.055 (0.200)	0.212 (0.101)∗	−1.520 (1.307)	−0.511 (0.477)	
-Non-contrib. pension-earner (NCP)	0.061 (0.141)	−0.042 (0.230)	−0.087 (0.123)	0.893 (1.566)	0.357 (0.574)	
Interviewed after July-07 (γ)	0.269 (0.122)∗	0.414 (0.348)	−0.128 (0.108)	−2.135 (1.121)	−1.678 (0.462)	∗∗∗
(Ref: Interviewed before)						

INTERACTIONS:						
After July−07 X NCP						
(Non-contribution pension) (δ)	−0.311 (0.215)	0.414 (0.348)	0.374 (0.217)†	−0.266 (2.133)	−0.095 (0.860)	
After July-07 X CP (Contribution pension) (λ)	−0.193 (0.194)	0.080 (0.330)	−0.118 (0.163)	0.429 (1.721)	0.202 (0.694)	

a Self-rated satisfaction with life is coded as health is coded as 5.Excellent, 4.Very good, 3.Good, 2.Fair, 1.Poor.

b Binary variables.

c †:*p* < 0.10; ∗:*p* < 0.05; ∗∗:*p* < 0.01; ∗∗∗:*p* < 0.001.

**Table 7:** Coefficients of random-effects Gaussian regressions for high density lipoprotein (HDL in mg/dl), total cholesterol (in mg/dl), triglycerides (in mg/dl), natural log of glycated hemoglobin (HbA_1C_ in %), and natural log of C-Reactive Protein (CPR in mg/dl), on social security status variables and control covariates.

Variables	HDL (mg/dl)	Total cholesterol (mg/dl)	Triglycerides (mg/dl)	Ln HbA_1C_(%) (×10^2^)	CRP (in mg/dl)
*Ordered probit regression with only non-contribution pension earners (Number of observations=5780; number of individuals=285)*					
(Ref: Interviewed before July 2007)					
Interviewed after June 2007	1.979 (1.044)†	−14.251 (4.058)∗∗∗	4.236 (7.054)	1.691 (1.334)	−0.145 (0.095)
*Ordered probit regression with all elderly population (Number of observations=3106; number of individuals=1553)*					
(Ref: Other social security status)					
-Contribution pension-earner (CP)	0.176 (0.732)	1.609 (2.233)	2.741 (4.967)	−0.435 (0.852)	0.023 (0.055)
-Non-contrib. pension-earner (NCP)	0.876 (0.141)	0.118 (2.675)	3.891 (5.947)	−0.575 (1.023)	−0.014 (0.066)
Interviewed after July−07 (γ)	2.890 (0.611)∗∗∗	−19.564 (1.955)∗∗∗	0.211 (4.213)	0.694 (0.774)	−0.080 (0.052)
(Ref: Interviewed before)					

INTERACTIONS:					
After July−07 X NCP					
(Non-contribution pension) (δ)	−1.151 (1.162)	2.590 (3.687)	5.217 (8.016)	0.662 (1.472)	−0.075 (0.099)
After July-07 X CP (Contribution pension) (λ)	−0.651 (0.938)	0.842 (2.975)	−3.588 (6.468)	0.015 (1.189)	0.049 (0.080)

Note: †:*p* < 0.10; ∗:*p* < 0.05; ∗∗:*p* < 0.01; ∗∗∗:*p* < 0.001.
